# The METS-IR is independently related to bone mineral density, FRAX score, and bone fracture among U.S. non-diabetic adults: a cross-sectional study based on NHANES

**DOI:** 10.1186/s12891-023-06817-9

**Published:** 2023-09-13

**Authors:** Bin Pu, Peng Gu, Dan Yue, Qiao Xin, WeiSong Lu, JiaSheng Tao, DaoZe Ke, Hui Chen, YangCheng Ma, WeiDong Luo

**Affiliations:** 1 Guangzhou University of Chinese Medicine, Guangzhou, Guangdong China; 2 Southwest Medical University, Luzhou, Sichuan China; 3 Jiangxi University of Chinese Medicine, Nanchang, Jiangxi China; 4 Luzhou Traditional Chinese Medicine Hospital, Luzhou, Sichuan China; 5 The First Affiliated Hospital, Guangzhou University of Chinese Medicine, Guangzhou, China

**Keywords:** METS-IR, Bone mineral density, FRAX score, Bone fractures, NHANES, Diabetic

## Abstract

**Aim:**

The purpose of this study was to investigate the association between the metabolic score for insulin resistance (METS-IR) and bone mineral density (BMD) in American non-diabetic adults.

**Methods:**

We conducted a cross-sectional study with 1114 non-diabetic adults from the National Health and Nutrition Examination Survey cycle (2013–2014). The associations between METS-IR and BMD of total femur and spine were assessed by the multiple linear regression and verified the non-linear relationship with a smooth curve fit and threshold effect model. Furthermore, we evaluated the relationship between METS-IR, FRAX score, and history of bone fractures.

**Results:**

We found that BMD of the total femur and spine increased by 0.005 g/cm^3^ and 0.005 g/cm^3^, respectively, for a one-unit increase of METS-IR in all participants. This positive association was more pronounced among higher METS-IR participants, and there was a non-linear relationship, which was more significant when the MTTS-IR_femur_ was < 41.62 or the METS-IR_spine_ was < 41.39 (β_femur_ = 0.008, β_spine_ = 0.011, all P < 0.05). We also found that METS-IR was positively correlated with both FRAX scores in all female participants. However, METS-IR was positively correlated only with the 10-year hip fracture risk score in male participants with fractures. No significant association between METS-IR and a history of bone fractures.

**Conclusions:**

In American non-diabetic adults, there is a correlation between elevated levels of METS-IR within the lower range and increased BMD as well as decreased risk of fractures, suggesting that METS-IR holds promise as a novel biomarker for guiding osteoporosis (OP) prevention. However, it is important to carefully balance the potential benefits and risks of METS-IR in OP.

## Introduction

OP is a chronic metabolic skeletal disorder characterized by decreased bone mineral density (BMD) and increased risk of fractures [1]. With the gradual aging of the population, osteoporosis (OP) has become a severe threat to public health [2]. About 1.5 million cases of osteoporotic fractures are reported worldwide annually [3]. The prevalence of OP is 16.0% in men aged 50 or above and 29.9% in postmenopausal women [4]. From the aspect of pathophysiology, OP is a complex disease determined by various genes and environmental factors [1]. In addition to uncontrollable risk factors such as race, female menopause, and aging, many controllable risk factors (such as low body weight, smoking, drinking, etc.) also play an essential role in the pathogenesis of OP [1]. Currently, there is no way to cure OP. Therefore, based on the controllable risk factors of osteoporosis, exploring ways to prevent and treat OP and reduce fracture risk is still a significant public health challenge today.

Metabolic syndrome (MetS) is a complex disorder characterized by a combination of various metabolic abnormalities, including central obesity, insulin resistance (IR), hypertension, dyslipidemia, and blood glucose instability [5]. IR, an essential component of metabolic syndrome, is a crucial mechanism in glucolipid metabolism [6]. IR is also a pathophysiological marker of many chronic diseases, including diabetes, cardiovascular disease, hypertension, and asthma [7–9]. In addition, previous studies have confirmed that IR is correlated with BMD and OP [10–12], but the results are inconsistent.

Hyperinsulinemic normoglycemic clamps (HECs) are currently the gold standard for assessing insulin sensitivity in peripheral tissues [13]. However, it is unsuitable for large-scale epidemiological studies and OP screening because of its invasiveness, complexity, and resource consumption. Therefore, in previous epidemiological studies, many non-invasive, easy-to-operate, and repeatable evaluation IR indicators have been developed, such as triglyceride glucose (TyG), TyG with body mass index (TyG-BMI), the ratio of triglyceride divided by high-density lipoprotein cholesterol (TG/HDL-C) and the metabolic score of insulin resistance (METS-IR). Their accuracy has been confirmed in the screening and diagnosis of IR [14–16]. Previous studies have indicated a correlation between IR and OP. However, currently, there is a lack of research investigating the relationship between METS-IR and BMD as well as OP. Therefore, we aimed to explore whether there was a clear correlation between METS-IR and BMD using the large sample size and representative samples of the NHANES database.

## Methods

### Data and sample sources

Data for this study were obtained from the National Health and Nutrition Examination Survey (NHANES). This is a nationally representative cross-sectional survey designed and conducted by the National Center for Health Statistics (NCHS). The NCHS Research Ethics Review Committee reviewed and approved the survey verifying that all participants provided informed consent. Detailed statistics can be accessed at https://www.cdc.gov/nchs/nhanes/.

**Fig. 1:**
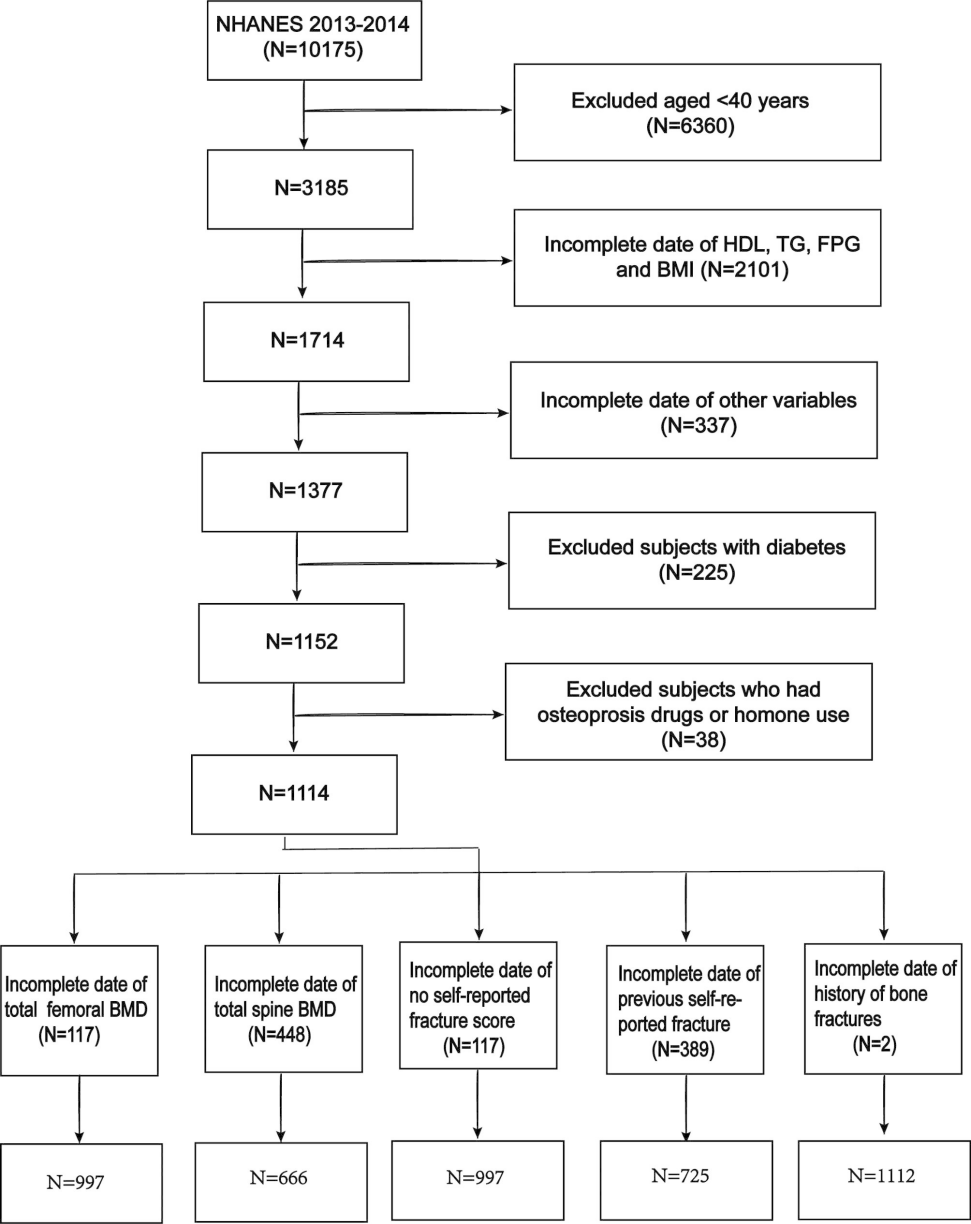
Flow chart of participants selection

This study uses the public data files of NHANES from 2013 to 2014 to construct a data set. Inclusion criteria include (1) participants ≥ 40 years old; (2) participants with complete HDL-C, TG, fasting plasma glucose (FPG), and BMI data; (3) participants with one of the four outcome indicators (total femoral BMD, total spinal BMD, fracture risk assessment tool (FRAX) score or previous fracture). Exclusion criteria included: (1) participants who have been treated for OP (who have been treated for osteoporosis); (2) prednisone or cortisone every day (prednisone or cortisone tablets almost every day for a month or more?); (3) participants with diabetes; (4) participants with missing data of other variables. Finally, out of 10,175 participants, a total of 1,114 participants were included in the study through strict eligibility criteria (Fig. 1).

### Exposure variable

Previous studies calculated METS-IR using participants’ BMI, HDL-C, TC, and FPG. METS-IR was calculated as follows: Ln [(2 × FPG (mg/dL) + TC (mg/dL)] × BMI (kg/m^2^) / {Ln [HDL-C (mg/dL)]} [15]. On the Modular Chemistry side of the DxC800, FPG was measured by an enzyme hexokinase method. Serum TC and HDL-C were measured using the Roche Modular P chemical analyzer and Roche Cobas 6000 chemical analyzer.

### Outcome variable

Total femoral BMD and total spinal BMD were determined by dual-energy X-ray absorptiometry (DXA) with rapid, easy-to-use, and low radiation exposure. The DXA inspection is performed by trained technicians using the Hollodge QDR-4500 A fan-beam densitometer (Hologic, Inc., Bedford, MA, USA) and the software version Apex3.2. For more information about the DXA exam, visit the NHANES website (https://wwwn.cdc.gov/nchs/nhanes/). The FRAX score was based on several fracture risk factors, including age, sex, weight, height, previous fracture, parental history of hip fracture, glucocorticoid use, rheumatoid arthritis, secondary osteoporosis, current smoking and alcohol consumption, and bone mineral density of the femoral neck. More information can be found on the FRAX website [17]. The previous fracture was by asking participants if their doctor had told them that they had suffered a fracture.

### Definition of other variables

The poverty income ratio (PIR) assesses the income situation. PIR < 1 is defined as poor, 1–3 is defined as near poor, and ≥ 3 is defined as not poor [18]. Menopause was assessed by women choosing menopause/change of life as an answer (“What is the reason that you have not had a period in the past 12 months?”) and.

choosing yes as an answer (Had both ovaries removed?) in the questionnaire. The participants answered hysterectomy (“What is the reason that you have not had a period in the past 12 months?“), or participants with missing data in the reproductive health questionnaire whose serum estradiol level < 30 pg/ml is defined as menopause [19].

### Covariates

We selected these covariates based on support from relevant literature and their associations with the estimated results or effect of interest, demonstrating changes of over 10% in the relationships [20–22]. The covariates included age, sex, race, education, marital status, alcohol consumption, smoking status, PIR, BMI, hypertension, serum creatinine (SCr), blood urea nitrogen (BUN), serum uric acid (SUA), total cholesterol (TC), TG, HDL-C, low-density lipoprotein cholesterol (LDL-C), PFG, 25 (OH) D, and serum calcium. Covariates were collected through family interviews, physical examinations, laboratory measurements, and questionnaires. For more details on data collection, visit https://wwwn.cdc.gov/nchs/nhanes/ContinuousNhanes/Default.aspx?BeginYear=2013.

### Statistical analyses

To account for oversampling in complex survey design, survey nonresponse, and poststratification, we used the 2-y sampling weight (WTMEC2YR) constructed by NHANES 2013–2014. Among the baseline features of all participants in the study, variables with continuous characteristics were expressed as means together with their standard deviations (mean ± SD), and categorical characteristics were expressed as percentages (%). For the preliminary analysis, weighted multiple linear regression determined the linear relationship between METS-IR and BMD, METS-IR and FRAX scores of different gender groups. Weighted multivariate logistic regression determined the association between METS-IR and a history of bone fractures. In model 1, no adjustment for covariates was made. Model 2 was adjusted for age and race. Model 3 was adjusted for age, race, education, marital status, PIR, smoking status, alcohol consumption, TG, LDL-C, Scr, SUA, BUN, and hypertension were adjusted. To further evaluate the relationship between METS-IR and total femoral BMD and total spinal BMD, smooth curve fitting (penalty spline method) and generalized additive model (GAM) regression were used. A likelihood ratio test calculated inflection points if a nonlinear relationship was identified.

All descriptive studies used a two-sided test with a significance level of P < 0.05 for significance test. All analyses were conducted using R (version 4.0.3) and EmpowerStats software (http://www.empowerstats.com). In addition, the sample size was based on the existing data, and the minimum sample size was not calculated in advance.

## Results

Based on the METS-IR quartile, the study participants’ baseline characteristics were shown in Table 1. The average age of the participants was 58.61 ± 12.21 years old, including 547 men (49.1%), 148 non-menopausal women (13.29%), and 419 postmenopausal women (37.61%). There were significant differences in sex, race, education, PIR, BMI, 25 (OH) D, Ca, TC, TG, HDL-C, LDL-C, FPG, Scr, SUA, and hypertension among different METS-IR groups. Interestingly, hypertension participants with lower incomes had significantly higher METS-IR. The opposite pattern was observed in education status. This is consistent with our previous research [23, 24].

**Table 1:** Baseline characteristic of the study population according to METS-IR.

Variables	Total (n = 1114)	Q1 (n = 279)	Q2 (n = 278)	Q3 (n = 278)	Q4 (n = 279)	P value
Age (years)	58.61 ± 12.21	59.16 ± 12.45	60.00 ± 12.39	58.00 ± 12.32	57.27 ± 11.55	0.053
Sex,n(%)						< 0.001
Men	547 (49.10%)	108 (38.71%)	149 (53.60%)	165 (59.35%)	125 (44.80%)	
Non-menopausal women	148 (13.29%)	44 (15.77%)	31 (11.15%)	33 (11.87%)	40 (14.34%)	
Menopausal women	419 (37.61%)	127 (45.52%)	98 (35.25%)	80 (28.78%)	114 (40.86%)	
Race/ethnicity, n (%)						< 0.001
Mexican American	135 (12.12%)	18 (6.45%)	29 (10.43%)	49 (17.63%)	39 (13.98%)	
Other Hispanic	89 (7.99%)	16 (5.73%)	21 (7.55%)	29 (10.43%)	23 (8.24%)	
Non-Hispanic White	544 (48.83%)	135 (48.39%)	135 (48.56%)	126 (45.32%)	148 (53.05%)	
Non-Hispanic Black	201 (18.04%)	45 (16.13%)	49 (17.63%)	50 (17.99%)	57 (20.43%)	
Other Race	145 (13.02%)	65 (23.30%)	44 (15.83%)	24 (8.63%)	12 (4.30%)	
Education level, n (%)						< 0.001
Under high school	240 (21.54%)	47 (16.85%)	64 (23.02%)	66 (23.74%)	63 (22.58%)	
High school or equivalent	226 (20.29%)	56 (20.07%)	50 (17.99%)	60 (21.58%)	60 (21.51%)	
Some College or AA degree	334 (29.98%)	64 (22.94%)	87 (31.29%)	86 (30.94%)	97 (34.77%)	
College Graduate or above	314 (28.19%)	112 (40.14%)	77 (27.70%)	66 (23.74%)	59 (21.15%)	
Marital status, n (%)						0.101
Live with someone	726 (65.17%)	170 (60.93%)	191 (68.71%)	191 (68.71%)	174 (62.37%)	
Live alone	388 (34.83%)	109 (39.07%)	87 (31.29%)	87 (31.29%)	105 (37.63%)	
PIR, n (%)						0.005
Poor	204 (18.31%)	46 (16.49%)	48 (17.27%)	48 (17.27%)	62 (22.22%)	
Near poor	415 (37.25%)	87 (31.18%)	103 (37.05%)	105 (37.77%)	120 (43.01%)	
Not poor	495 (44.43%)	146 (52.33%)	127 (45.68%)	125 (44.96%)	97 (34.77%)	
Smoking status, n (%)						0.102
Never	590 (52.96%)	164 (58.78%)	150 (53.96%)	137 (49.28%)	139 (49.82%)	
Former	290 (26.03%)	58 (20.79%)	65 (23.38%)	84 (30.22%)	83 (29.75%)	
Current	234 (21.01%)	57 (20.43%)	63 (22.66%)	57 (20.50%)	57 (20.43%)	
Alcohol consumption, n (%)						0.715
Yes	794 (71.27%)	192 (68.82%)	202 (72.66%)	202 (72.66%)	198 (70.97%)	
No	320 (28.73%)	87 (31.18%)	76 (27.34%)	76 (27.34%)	81 (29.03%)	
Hypertension, n (%)						< 0.001
Yes	497 (44.61%)	89 (31.90%)	119 (42.81%)	133 (47.84%)	156 (55.91%)	
No	617 (55.39%)	190 (68.10%)	159 (57.19%)	145 (52.16%)	123 (44.09%)	
BMI (kg/m2)	28.86 ± 6.80	22.24 ± 2.30	26.22 ± 2.01	29.55 ± 2.50	37.43 ± 6.66	< 0.001
25(OH)D (nm/L)	9.40 ± 0.34	9.45 ± 0.35	9.41 ± 0.33	9.38 ± 0.30	9.36 ± 0.38	0.004
Calcium (mg/dL)	69.37 ± 28.11	76.47 ± 30.85	70.72 ± 27.85	67.27 ± 25.70	63.02 ± 26.13	< 0.001
TC (mg/dL)	195.32 ± 40.69	199.21 ± 38.58	195.25 ± 44.85	196.32 ± 39.87	190.50 ± 38.90	0.024
TG (mg/dL)	112.70 ± 64.59	78.71 ± 37.79	99.47 ± 51.75	124.28 ± 62.56	148.32 ± 77.22	< 0.001
HDL-C (mg/dL)	55.85 ± 16.96	70.85 ± 18.64	56.55 ± 12.93	49.74 ± 11.46	46.24 ± 12.28	< 0.001
LDL-C (mg/dL)	116.93 ± 36.47	112.63 ± 34.81	118.78 ± 40.11	121.73 ± 35.37	114.60 ± 34.85	0.021
FPG (mg/dL)	103.35 ± 20.18	96.74 ± 10.63	101.92 ± 21.54	104.68 ± 16.87	110.07 ± 26.05	< 0.001
SCr (mg/dL)	0.90 ± 0.26	0.85 ± 0.21	0.94 ± 0.32	0.92 ± 0.24	0.89 ± 0.26	< 0.001
SUA (mg/dL)	5.50 ± 1.37	4.89 ± 1.29	5.36 ± 1.30	5.74 ± 1.30	6.00 ± 1.34	< 0.001
BUN (mg/dL)	13.60 ± 5.32	13.20 ± 4.53	14.33 ± 5.98	13.29 ± 4.83	13.57 ± 5.76	0.088

PIR, poverty income ratio; BMI, body mass index; TC, total cholesterol; TG, triglyceride; HDL-C, high-density lipoprotein cholesterol; LDL-C, low-density lipoprotein cholesterol; FPG, fasting plasma glucose; SCr, serum creatinine; SUA, serum uric acid; BUN, blood urea nitrogen; METS-IR, metabolic score for insulin resistance; Ql, Q2, Q3, and Q4 are quartiles of the metabolic score for insulin resistance (METS-IR). PIR < 1 is defined as poor, 1–3 is defined as near poor, and ≥ 3 is defined as not poor

### Association between METS-IR and BMD

Table 2 showed the results of the multivariate regression analysis. After controlling for different potential confounders, all four models showed a positive correlation between METS-IR and BMD levels. When METS-IR was used as a continuous variable in the fully adjusted model (model 3), for every unit increase in METS-IR, the total femur BMD and total spine BMD increased 0.005 g/cm^3^, 0.005 g/cm^3^, respectively. When METS-IR was converted to classification variable according to quartile, compared with that of participants with lower METS-IR Q1, the adjusted βvalues of METS-IR and total femoral BMD in Q2, Q3 and Q4 were 0.042, 0.086, and 0.124 respectively. In the complete adjustment model, those of total spine BMD were 0.059, 0.105, and 0.142 (all P<0.05). In addition, the total femoral BMD and total spinal BMD levels of the participants showed an upward trend with the increase of METS-IR (P for trend < 0.001) (Table 2).

**Table 2:** Multivariable-adjust β and 95%CI of the METS-IR quartiles associated with total femur and total spine BMD.

		Model 1, β (95%CI)	Model 2, β (95%CI)	Model 3, β (95%CI)
**Total femur** **(n = 997)**	METS-IR	0.006 (0.005, 0.007)	0.005 (0.005, 0.006)	0.005 (0.004, 0.006)
	**Quintiles of METS-IR**			
	Q1(20.1-33.86)	Reference	Reference	Reference
	Q2(33.87–39.46)	0.065 (0.040, 0.089)	0.045 (0.023, 0.067)	0.042 (0.019, 0.065)
	Q3(39.47–47.35)	0.127 (0.102, 0.151)	0.098 (0.076, 0.121)	0.086 (0.062, 0.110)
	Q4(47.37–87.99)	0.155 (0.131, 0.179)	0.131 (0.109, 0.154)	0.124 (0.099, 0.149)
	P for trend	< 0.001	< 0.001	< 0.001
	Increase per one	0.007	0.006	0.005
**Total spine** **(n = 666)**	METS-IR	0.006 (0.005, 0.007)	0.005 (0.004, 0.006)	0.005 (0.004, 0.006)
	**Quintiles of METS-IR**			
	Q1(20.1-33.54)	Reference	Reference	Reference
	Q2(33.56–39.87)	0.068 (0.035, 0.101)	0.059 (0.028, 0.090)	0.059 (0.027, 0.091)
	Q3(39.95–47.35)	0.123 (0.090, 0.157)	0.109 (0.077, 0.141)	0.105 (0.070, 0.140)
	Q4(47.37–86.88)	0.165 (0.132, 0.198)	0.147 (0.114, 0.179)	0.142 (0.106, 0.178)
	P for trend	< 0.001	< 0.001	< 0.001
	Increase per one	0.007	0.006	0.006

Model 1: No covariates were adjusted. Model 2: Age, Race were adjusted. Model 3: Age, Race, Education, Marital status, PIR, Smoking, Alcohol consumption, Hypertension, Calcium, 25(OH)D, TC, LDL-C, SCr, SUA and BUN were adjusted in the model

Furthermore, we conducted a subgroup analysis according to gender. We found that METS-IR was positively correlated with total femoral BMD and total spinal BMD in men, non-menopausal and postmenopausal women (all P<0.05) (Table 3).

**Table 3:** Adjusted regression coefficients (S.E.) for differences in total femur and total spine BMD relative to a one unit increase in METS-IR.

BMD(g/cm^3^)		Men		Women			
				Non-menopausal		Menopausal	
		β (95%CI) P		β (95%CI) P		β (95%CI) P	
**Total femur**	Population	502		131		364	
	Model1	0.005 (0.004, 0.006)	< 0.001	0.006 (0.005, 0.008)	< 0.001	0.006(0.005, 0.007)	< 0.001
	Model2	0.005 (0.004, 0.006)	< 0.001	0.006 (0.005, 0.008)	< 0.001	0.005(0.004, 0.006)	< 0.001
	Model3	0.007 (0.004, 0.009)	< 0.001	0.005 (0.002, 0.009)	0.006	0.003 (0.001, 0.006)	0.004
**Total spine**	Population	314		124		228	
	Model1	0.004 (0.002, 0.006)	< 0.001	0.004 (0.002, 0.005)	< 0.001	0.007 (0.005, 0.009)	< 0.001
	Model2	0.004 (0.002, 0.005)	< 0.001	0.004 (0.002, 0.006)	< 0.001	0.007 (0.005, 0.008)	< 0.001
	Model3	0.007 (0.004, 0.010)	< 0.001	0.005 (0.001, 0.009)	0.028	0.007 (0.003, 0.011)	< 0.001

Model 1: No covariates were adjusted. Model 2: Age, Race were adjusted. Model 3: Age, Race, Education, Marital status, PIR, Smoking, Alcohol consumption, Hypertension, Calcium, 25(OH)D, TC, LDL-C, SCr, SUA and BUN were adjusted in the model

**Fig. 2:**
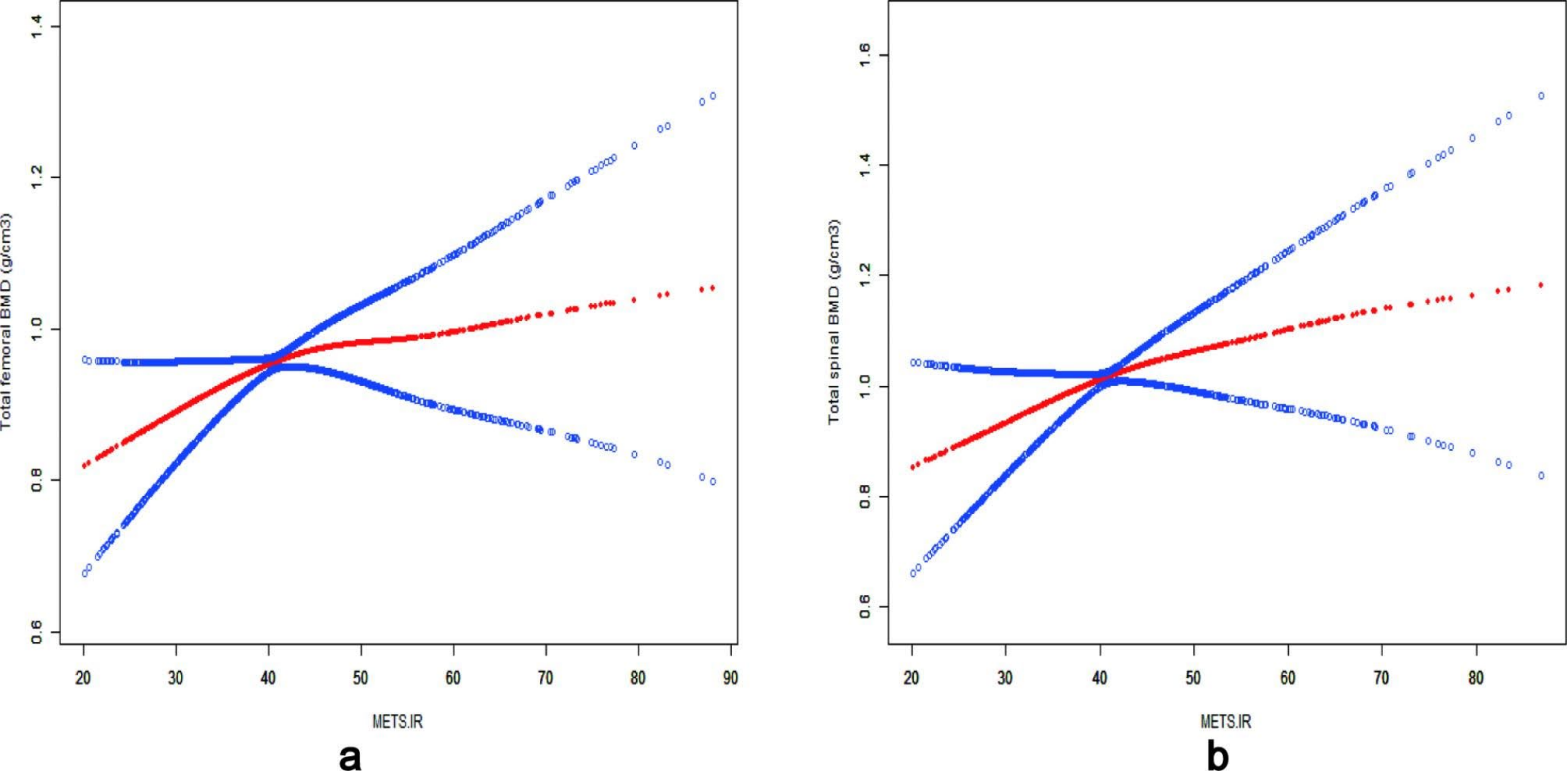
**(a)** The association between METS-IR and total femoral BMD. **(b)** The association between METS-IR and total spine BMD. Solid red line represents the smooth curve fit between variables. Blue bands represent the 95% of confidence interval from the fit. Sex, Age, Race, Education, Marital status, PIR, Smoking, Alcohol consumption, Hypertension, Calcium, 25(OH)D, TC, LDL-C, SCr, SUA and BUN were adjusted in the model

Furthermore, the threshold effect is analyzed. The threshold effect model showed that when METS ≤ 41.62, the positive correlation between METS-IR and total femoral BMD was more significant in American non-diabetic adults (β = 0.008, P = 0.020). When METS ≤ 41.39, the positive correlation between METS-IR and total spine BMD was more significant in American non-diabetic adults (β = 0.011, P = 0.024). (Table 4; Fig. 2)

**Table 4:** The nonlinear relationship between METS-IR and total femoral BMD and total spinal BMD.

	Total femoral BMD		Total spine BMD	
	β (95% CI)	P-value	β (95% CI)	P-value
Model I: univariate linear regression	-0.001 (-0.006, 0.004)	0.726	0.001 (-0.006, 0.008)	0.687
Model II: two-piecewise regression model				
Inflection point (K)	41.62		41.39	
< K point effect 1	0.008 (0.001, 0.014)	0.020	0.011 (0.001, 0.020)	0.024
> K point effect 2	0.002 (-0.003, 0.007)	0.400	-0.006 (-0.010, -0.002)	0.004
Log-likelihood ratio test		< 0.001		0.003

Sex, Age, Race, Education, Marital status, PIR, Smoking, Alcohol consumption, Hypertension, Calcium, 25(OH)D, TC, LDL-C, SCr, SUA and BUN were adjusted in the model

### Association between METS-IR and FRAX score

Table 5 showed the linear regression coefficient (standard error) of a one-unit increase in the FRAX score (hip fracture and major osteoporotic fracture score) relative to the METS-IR. According to the medical history and DXA measurement, the results showed that METS-IR was positively correlated with both FRAX scores in all participants. After stratification by gender, METS-IR was positively correlated with both FRAX scores in all female participants. However, METS-IR was positively correlated only with the 10-year hip fracture risk score in male participants with fractures.

**Table 5:** Linear regression coefficients (standard error) for differences in FRAX scores (hip fracture and major osteoporotic fracture score) relative to a one unit increase in METS-IR.

Variable	Total		Men		women			
					Non-menopausal		Menopausal	
	β (SE)	P	β (SE)	P	β (SE)	P	β (SE)	P
No self-reported fracture after age 20 and no vertebral fracture measured by DXA								
Population	725		364		107		254	
10-year hip fracture risk score	-0.021(0.006)	0.001	-0.009(0.005)	0.091	-0.006(0.002)	0.014	-0.047(0.017)	0.005
10-year major osteoporotic fracture risk score	-0.040(0.011)	< 0.001	-0.017(0.009)	0.061	-0.029(0.009)	0.002	-0.068(0.027)	0.012
Previous self-reported fracture after age 20 or vertebral fracture measured by DXA								
Population	997		502		131		364	
10-year hip fracture risk score	-0.039(0.008)	< 0.001	-0.023(0.008)	0.005	-0.035(0.010)	< 0.001	-0.063(0.019)	0.001
10-year major osteoporotic fracture risk score	-0.059(0.015)	0.001	-0.026(0.016)	0.121	-0.074(0.020)	< 0.001	-0.099(0.031)	0.002

Adjusted for model 3: Age, Race, Education, Marital status, PIR, Smoking, Alcohol consumption, Hypertension, Calcium, 25(OH)D, TC, LDL-C, SCr, SUA and BUN. SE, Standard Error

### The association between METS-IR and a history of bone fractures

With a fully adjusted model 3, we evaluated the relationship between a history of bone fractures and METS-IR after stratification by gender (Table 6). The results showed METS-IR increased by 1 unit, and all types of fracture risk decreased by 2% (95% CI = 0.96-1.00; P = 0.048)in males. The METS-IR increased by 1 unit, and all types of fracture risk increased by 2% (95% CI = 1.00-1.05; P = 0.030) in postmenopausal females. No positive results were found in other subgroups.

**Table 6:** Associations between history of bone fractures and a one unit increase in METS-IR in logistic regression models

	All types of fracture		Spine fracture		Hip fracture		Wrist fracture		Other fracture	
	OR (95% CI)	P value	OR (95% CI)	P value	OR (95% CI)	P value	OR (95% CI)	P value	OR (95% CI)	P value
Men	0.98 (0.96, 1.00)	0.048	0.99 (0.94, 1.04)	0.750	1.01 (0.88, 1.16)	0.916	0.97 (0.93, 1.02)	0.216	0.98 (0.96, 1.00)	0.088
Non-menopausal women	1.00 (0.94, 1.06)	0.920	1.40 (0.00, Inf)	1.000	0.89 (0.00, Inf)	1.000	0.82 (0.00, Inf)	1.000	1.02 (0.96, 1.09)	0.539
Menopausal women	1.02 (1.00, 1.05)	0.030	2.57 (0.00, Inf)	1.000	1.05 (0.97, 1.14)	0.230	1.02 (0.98, 1.06)	0.309	1.02 (1.00, 1.05)	0.088

Adjusted for model 3: Age, Race, Education, Marital status, PIR, Smoking, Alcohol consumption, Hypertension, Calcium, 25(OH)D, TC, LDL-C, SCr, SUA and BUN. OR: odds ratio

## Discussion

This is the first large-scale cross-sectional study using NHANES data to confirm the association between METS-IR, BMD, and FRAX scores. The study found that total femur BMD and spine BMD increased by 0.005 g/cm^3^ and 0.005 g/cm^3^ for a one-unit increase of METS-IR in American non-diabetic adults. This positive association persisted whether METS-IR was used as a continuous variable or quartiles were converted to categorical variables. It also suggested that the statistical difference in this association was more pronounced at higher METS-IR. Furthermore, similar results were found in METS-IR and FRAX scores among U.S. non-diabetic women. However, there is no significant correlation between a history of bone fractures and METS-IR. The dose-response relationship between METS-IR and total femoral BMD was also tested, and the threshold effect of METS-IR was 41.62. Compared with the left side of the inflection point, when the METS-IR was 41.62, the total femoral BMD increased with the increase of METS-IR (β = 0.008, 95%CI: 0.001–0.014). However, when the METS-IR is 41.62, this trend gradually becomes stable compared to the right side of the inflection point (β = 0.002, 95%CI: -0.003-0.007). Similar results were found in the dose-response relationship between METS-IR and total spinal BMD.

At present, the clinical diagnosis of OP is mainly through DXA [25]. The risk of OP can be evaluated by HDL-C and BMI. DXA is relatively expensive, has radiation and can only reflect the static, and local BMD of the patient [26]. Using laboratory indexes such as HDL-C and BMI [27] alone to predict the risk of OP has low sensitivity and specificity. Therefore, it is crucial to explore a more simple, economical, and accurate method to predict the risk of OP in ordinary people.

METS-IR was first reported in 2018 and was considered a reliable and intuitive IR prediction indicator [15]. It does not depend on insulin tests but on laboratory tests (such as lipid and blood sugar) and BMI, which is easily obtained in primary medical institutions [15, 28]. Compared with other IR indexes (TyG, TG/HDL-C), it considered the effects of BMI and other lipid types on bone metabolism. Therefore, METS-IR is more comprehensive in evaluating metabolic status and is recognized as an effective index for IR estimation in the Chinese population [24, 29–31].

The correlation between IR and BMD has been confirmed in previous studies, but the results are not consistent. A cross-sectional study of postmenopausal women in Tunisia by Cherif et al. [10] found that HOMA-IR was positively correlated with BMD of the left femur and total hip. Napoli et al. [32] found a positive correlation between IR and BMD in a prospective study of 2398 non-diabetic elderly. Yoon et al. [11] found that the TyG index was negatively correlated with femoral neck BMD in non-diabetic men and postmenopausal women over 50 in a cohort study of 4810 non-diabetic Koreans. Zhou et al. [12] found that the increase in HOMA-IR level was related to the increase of hip BMD in 7,170 American adults. However, no causal relationship was found between IR and BMD in a Mendelian randomized study of European adults. In addition, numerous studies [33–36] have proved that the indexes used to calculate METS-IR are significantly correlated with BMD. Therefore, we used NHANES 2013–2014 data to conduct this large cross-sectional study and evaluated the correlation between METS-IR and BMD and FRAX scores in American non-diabetic adults for the first time. The results showed that METS-IR was positively correlated with total femur BMD and spine BMD in all participants. METS-IR was positively correlated with both FRAX scores in women.

The contradictory findings may be attributed to the involvement of different study populations or the utilization of diverse methods for assessing IR. Based on the population of this study (the U.S. non-diabetic adults) and the IR assessment method (METS-IR), we believe that the possible mechanism of METS-IR affecting BMD and OP is as follows. Firstly, IR promotes insulin secretion, leading to hyperinsulinemia and increased BMD. Insulin plays a crucial role in the skeletal system by stimulating osteoblast proliferation, inhibiting osteoclast activity, and acting as a synthetic metabolite [37]. In the state of IR, insulin secretion rises to compensate for the resistance exhibited by skeletal muscles, adipose tissue, and the liver, resulting in hyperinsulinemia. Consequently, IR stimulates insulin secretion, further augmenting bone mass. In addition, the synergistic effect of excessive insulin and other synthetic metabolic hormones (parathyroid hormone, insulin-like growth factor) can also lead to BMD increase [38, 39]. Secondly, IR may influence bone metabolism by modulating inflammatory responses and estrogen levels. According to Wang et al. [40] the relationship between IR and OP is non-linear, exhibiting a threshold effect. Our study results confirm this perspective. When METS-IR < 41.62 or 41.39, increasing IR levels are associated with a reduced risk of OP in non-diabetic adults. However, when METS-IR ≥ 41.62 or 41.39, the protective effect of IR on bone diminishes. This effect could be attributed to increased pro-inflammatory cytokines and oxidative stress, as well as decreased estrogen levels, which adversely affect bone health and nullify the protective effect of IR [41, 42].

The primary strength of this study lies in its pioneering use of Mets-IR to assess the correlation between bone density in non-diabetic adults and the risk of OP, thereby opening up new avenues for investigating the link between insulin resistance and OP risk. This research has the potential to enhance the predictive biological indicators of OP risk to some extent and provide valuable insights for the screening, prevention, and treatment of osteoporosis in primary healthcare settings. However, we also acknowledge the limitations of this study. Firstly, our study was a cross-sectional study using the NHANES database, which could not determine the causal relationship between METS-IR and BMD; Second, all participants in this study were American residents, and this conclusion may not apply to all populations; Finally, collecting questionnaire data through questionnaires and interviews may lead to recall bias and affect the study’s conclusions. Despite these limitations, this study strongly proposed a new index to prevent OP and proved the relationship between METS-IR and BMD.

## Conclusion

In American non-diabetic adults, there is a correlation between elevated levels of METS-IR within the lower range and increased BMD as well as decreased risk of fractures, suggesting that METS-IR holds promise as a novel biomarker for guiding OP prevention. However, caution is warranted in balancing the potential benefits and risks of METs-IR in OP management. Further in-depth research and exploration are necessary to comprehensively understand the relationship between insulin resistance, bone density, and fracture risk.

## Data Availability

Some or all data generated or analyzed during this study are included in this published article or in the data repositories listed in References. NHANES data is available publically at https://wwwn.cdc.gov/nchs/nhanes/Default.aspx.

## List of abbreviations

METS-IR: metabolic score for insulin resistance
BMD: bone mineral density
NHANES: National Health and Nutrition Examination Survey
OP: osteoporosis
MetS: Metabolic syndrome
IR: insulin resistance
HECs: Hyperinsulinemic normoglycemic clamps
TyG: triglyceride glucose
TG: triglyceride
HDL-C: high-density lipoprotein cholesterol
BMI: body mass index
NCHS: National Center for Health Statistics
FPG: fasting plasma glucose
FRAX: fracture risk assessment tool
DXA: dual-energy X-ray absorptiometry
PIR: poverty income ratio
SCr: serum creatinine
BUN: blood urea nitrogen
SUA: serum uric acid
TC: total cholesterol
LDL-C: low-density lipoprotein cholesterol
GAM: generalized additive model
